# A combination of PD-1 and TIGIT immune checkpoint inhibitors elicits a strong anti-tumour response in mesothelioma

**DOI:** 10.1186/s13046-025-03314-w

**Published:** 2025-02-12

**Authors:** Huaikai Shi, Ta-Kun Yu, Ben Johnson, Sakthi Priya Selvamani, Ling Zhuang, Kenneth Lee, Sonja Klebe, Samuel Smith, Kirby Wong, Kate Chen, Georgina Clark, Emma M. Rath, Holly Pearson, David Gallego Ortega, Anthony Linton, Steven Kao, Pablo Silveira, Yuen Yee Cheng

**Affiliations:** 1 Asbestos and Dust Disease Research Institute (ADDRI), 3 Hospital Road, Sydney, NSW, Concord, NSW 2139 Australia; 2 Sydney Medical School, University of Sydney, Sydney, NSW 2050 Australia; 3 SydPath, St. Vincent Health, Darlinghurst, Sydney, NSW 2010 Australia; 4 Institute for Biomedical Materials & Devices (IBMD), Faculty of Science, The University of Technology Sydney, Ultimo, NSW, 15 Broadway, Ultimo, NSW 2007 Australia; 5 Pathology, Flinders Health and Medical Research Institute, Flinders University, Bedford Park, South 5042 Australia; 6 Department of Medical Oncology, Chris O’Brien Lifehouse, Sydney, NSW 2050 Australia; 7 Department of Radiology, Royal Prince Alfred Hospital, Sydney, 2050 Australia; 8 Dendritic Cell Research Group, ANZAC Research Institute, Sydney, NSW 2139 Australia; 9 Victor Chang Cardiac Research Institute, 405 Liverpool St, Darlinghurst, NSW 2010 Australia; 10 School of Clinical Medicine, Faculty of Medicine and Health, UNSW Sydney, Kensington, NSW Australia; 11 Single Cell Genomics Facility, School of Biomedical Engineering, Faculty of Engineering and IT, The University of Technology Sydney, Ultimo, NSW 2007 Australia; 12 School of Clinical Medicine, Faculty of Medicine and Health, University of New South Wales, Kensington, NSW 2033 Australia; 13 The Kinghorn Cancer Centre, Garvan Institute of Medical Research, 380 Victoria St. Darlinghurst, Sydney, NSW 2010 Australia

## Abstract

**Background:**

Finding effective and curative treatment for mesothelioma remains challenging. While the introduction of immunotherapy combinations using ipilimumab (anti-CTLA-4) and nivolumab (anti-PD-1) have offered hope for some patients, a large proportion of mesothelioma cases, particularly the epithelial subtype, have minimal benefit from this.

**Methods:**

Our study was inspired by the results of the AdvanTG-105 phase I clinical trial, which showed partial response with anti-TIGIT/PD-1 treatment in two epithelioid mesothelioma patients. Here, we conducted a comprehensive in vivo experiment involving eight animal treatment groups administered with either PBS (control group), cisplatin/pemetrexed, anti-PD-1, anti-PD-1 + anti-CTLA-4, anti-TIGIT, anti-PD-1 + anti-TIGIT, anti-PD-1 + anti-CTLA-4 + anti-TIGIT, and cisplatin/pemetrexed + anti-PD-1 + anti-TIGIT.

**Results:**

Our results indicate that animals receiving anti-PD-1 + TIGIT exhibited a superior anti-tumour response, with 90% of the treatment group exhibiting an objective response, compared to 60%, 20% and 40% for the standard-of-care anti-PD-1 + CTLA-4, single-agent anti-PD-1 and cisplatin/pemetrexed treatment groups, respectively. Animals receiving anti-PD-1 + TIGIT displayed a significantly reduced average tumour size, with improved weight and survival rates, and fewer adverse effects than those receiving anti-PD-1 + CTLA-4 treatment. Anti-PD-1 + TIGIT-treated animals achieved complete tumour regression, with heightened effector CD8 + T cell and NK cell activity, remaining tumour-free for over 300 days without immune-related adverse events. After initial tumour elimination, anti-PD-1 + TIGIT-treated animals showed no tumour regrowth in the rechallenge experiment.

**Conclusion:**

These findings provide rationale for the development of an anti-PD-1 + TIGIT combination immunotherapy trial for mesothelioma patients.

**Graphical Abstract:**

Top) The comparison of standard-of-care treatment and anti-TIGIT novel combination treatment in the mesothelioma animal models, with an example of response treated with tislelizumab and ociperlimab in a pleural mesothelioma patient in the AdvanTIG-105 study. The number of animals/patients treated and the number of treatment responders are presented. Bottom) Schematic illustration of anti-tumour immune response at the cellular level induced by anti-PD-1/TIGIT checkpoint blockade for efficient cancer immunotherapy.

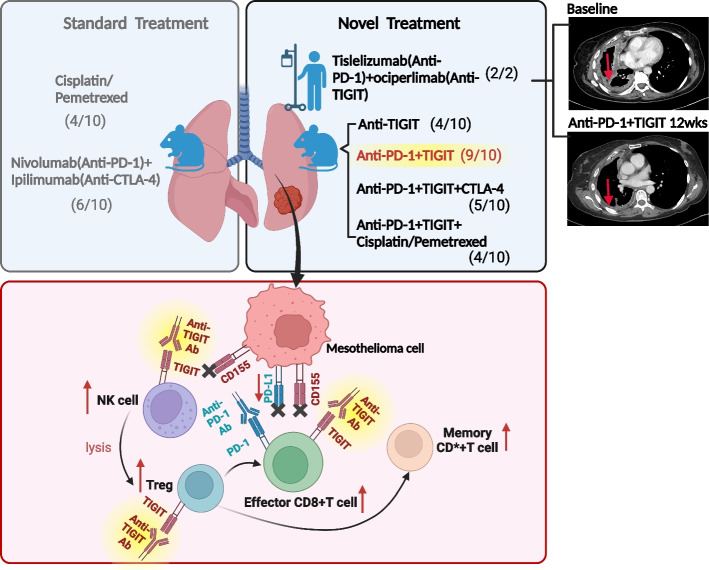

**Supplementary Information:**

The online version contains supplementary material available at 10.1186/s13046-025-03314-w.

## Introduction

Pleural mesothelioma (PM) is a highly aggressive, fast-growing asbestos-induced cancer affecting the mesothelial lining of the lung, with poor 5-year survival [1–3]. The single-agent anti-PD-1 antibody, pembrolizumab, was reported to have a partial response of 20% in PM patients [4, 5]. With the addition of ipilimumab (anti-CTLA-4) to nivolumab (anti-PD-1), a response was seen in up to 40% of PM patients [6]. Nivolumab and ipilimumab combination immunotherapy represents a significant improvement in the treatment of non-epithelioid PM patients, given that it enhances median survival by more than two-fold compared to chemotherapy-treated patients [6]. However, the epithelioid PM subgroup patients experienced only a modest benefit with nivolumab/ipilimumab compared to chemotherapy, with a median survival gain of 1.5 months [6]; arguably not clinically meaningful. Moreover, checkpoint inhibitors that target CTLA-4 are more frequently associated with severe immune-related adverse events [7–9]. Therefore, the development of alternative checkpoint blockade strategies to combine with anti-PD-1/PD-L1 warrants further attention, particularly for epithelioid subtype PM.

T cell immunoreceptor with Ig and ITIM domains (TIGIT) is a promising new target in cancer immunotherapy that is currently under investigation [10–13]. TIGIT is an immunosuppressive receptor expressed on several types of lymphocytes, including activated T cells, natural killer (NK) cells, and regulatory T (T reg) cells [12]. While single TIGIT blockade has demonstrated poor anti-tumour efficacy, co-blockade of TIGIT and PD-1/PD-L1 has shown significant tumour rejection (complete regression after treatment and the absence of recurrent tumour for the entire follow-up period) and prolonged overall survival in several preclinical mouse models [10]. It is currently unknown whether anti-TIGIT blockade alone or in combination with other immune checkpoint inhibitors (ICIs) is effective in PM.

In the present study, we describe the two epithelioid PM patients who participated in the AdvanTIG-105 study and exhibited a partial response (PR) following treatment with tislelizumab (anti-PD-1 antibody) and ociperlimab (anti-TIGIT antibody). We then demonstrate in a mouse model of mesothelioma that the combination of anti-PD-1 and anti-TIGIT elicits an enhanced anti-tumour response compared to clinical standard-of-care therapies (anti-PD-1 + CTLA-4 combination and chemotherapy). Additionally, long-term immune memory effect was observed in animals that received anti-PD-1 and anti-TIGIT treatment. No promising circulating immune markers were found, but increased T reg, effector CD8 + T cells and NK cells in the tumour microenvironment were associated with better anti-tumour response in anti-PD-1 and anti-TIGIT treated mice. Our study provides a biological rationale to pursue a clinical trial utilising the anti-PD-L1 and anti-TIGIT treatment strategy in epithelioid mesothelioma.

## Methods

### Anti-TIGIT/PD-1 combination treatment in PM patients

AdvanTIG-105 trial is an open-label, multicentre, phase 1, first-in-human dose escalation study of the anti-TIGIT monoclonal antibody ociperlimab in combination with tislelizumab (PD-1 antibody) in patients with advanced solid tumours [14]. Out of the 32 patients that were enrolled in the study, two ICI-naïve patients had epithelioid PM.

### Mesothelioma tumour model and treatment

The effect of tumour growth of different combinations of immunotherapy and/or chemotherapy was evaluated in an immunocompetent CBA mice (H-2^ k^) model. Seven- to eight-week-old female animals were purchased from the Animal Resource Centre (WA, Australia). Animals were housed with free access to water in the specific pathogen-free translation research facility (TRF) at the ANZAC Research Institute (Concord, NSW, Australia). The environment was closely controlled at 24–26 and 44–46% humidity under a 12:12 h light–dark cycle with lights on at 6 am. All protocols were approved by the Sydney Local Health District Animal Welfare Committee (Protocol no. 2019/023) under the Australian National Health and Medical Research Council guidelines for animal experimentation.

### Tumour cell lines and tumour inoculation

A mouse epithelioid PM cell line (AC29) was purchased from Cellbank Australia [15] and was stably transfected with a pGL-51lu luciferases construct. This stable cell line enabled the visualisation of tumours via in situ bioluminescence detection in live animals using the IVIS spectrum in vivo imaging system (PerkinElmer). AC29 cells were cultured at 37 °C in 5% CO2 in RPMI 1640 medium supplemented with 10% Foetal Calf Serum and 2% Geneticin (Gibco). For tumour inoculation, AC29 cells were taken out of culture using 0.05% Trypsin (Gibco), washed once with PBS and resuspended in culture media. Mice were intraperitoneal (i.p.) injected with 1 million AC29 cells in 200 µl injection volume. Mice were considered as ‘tumour-bearing’ once tumour nodules were visualised by IVIS. Mice were monitored daily and euthanised in accordance with the Sydney Local Health District (SLHD) animal ethics (2019/023).

### In vivo treatment

Animals were randomly separated into eight treatment groups: 1. PBS control; 2. cisplatin/pemetrexed; 3. Anti-PD1 alone; 4. Anti-PD1 + anti-CTLA-4; 5. Anti-TIGIT; 6. Anti-TIGIT + anti-PD-1; 7. Anti-TIGIT + anti-PD-1 + anti-CTLA-4; 8. Anti-TIGIT + anti-PD-1 + cisplatin/pemetrexed.

Mice were intraperitoneally (i.p.) injected with 1 million AC29 cells in a 200 µl injection volume. Mice were considered as ‘tumour-bearing’ once tumour nodules were clearly visualised by IVIS on Day 7. For single or combination immune-checkpoint blockade treatments, anti-PD-1(RMP1-14) (200 µg/mouse), anti-CTLA-4(9D9)(200 µg/mouse) and anti-TIGIT(1G9)(200 µg/mouse) (BioXcell, low endotoxin azide-free, using the recommended dose from the manufacturer (Supplementary Table 1). Antibodies were i.p. injected weekly at Day 7, 14, 21, 28.

For the chemotherapy treatment group, cisplatin (0.32 mg/mouse, Merck) and pemetrexed (2.56 mg/mouse, Merck) were administrated singularly or in combination with immunotherapy via intravenous (i.v.) injection. Tumour response in mice was defined by reduced tumour volume by Day 28 without subsequent regrowth of the tumour. The response rate in mice was defined as the number of mice that exhibited an anti-tumour response divided by the total number of treated mice in that group.

### Rechallenge of cured mice with tumour cells

Long-term surviving animals without detectable tumour were continually monitored weekly after treatment with anti-PD-1 plus TIGIT antibodies. At Day 300, cured mice (*N* = 9) were re-challenged with AC29 mesothelioma cells (1 million, i.p.). At the same time, five sex-matched naive CBA mice were inoculated with the same number of AC29 cells.

### Mouse tissue processing

Tumour, liver, lung, spleen and kidney tissue were harvested from all mice and fixed in 10% buffered formalin (Merck) for 24 h prior to tissue processing and paraffin embedding.

### Flow cytometry

Single-cell suspensions were prepared from fresh mouse spleen and tumour tissue by mechanical disruption through 70-µm (BD, spleen) and 40-µm (Edwards, tumour) cell trainers in RMPI 1640 media supplemented with 2% heat-inactivated fetal bovine serum (ThermoFisher, 2% FCS/RPMI 1640). Red blood cells were removed from spleens with 1X RC lysis buffer (eBioscience). Harvested cells (2 × 10^6^) were resuspended in diluted 100 μl Zombie UV solution (1:100, Biolegend) and incubated at room temperature (in the dark) for 30 min. Cells were then treated with Fc receptor binding inhibitor and stained with antibodies listed in Supplementary Table 1. Extracellular staining was performed in FACS buffer (0.1% BSA, 2 mM EDTA, PBS) for 30 min at room temperature using CD107A (1:50), CD25 (1:50), TIGIT (1:50), CD45 (1:100), CD49b (1:200), CD4 (1:200), CD62L (1:200), CD8a (1:200), CD279 (1:50), CD3(1:200), CD44 (1:200), CD152 (1:50). For intracellular staining, a Foxp3 fixation/permeabilization kit (Biolegend) was used. In brief, cells were incubated in fixation buffer at room temperature (in the dark) for 60 min, and then stained with FOXP3 (1:50) in permeabilization buffer for 30 min at room temperature. Data was acquired on the BD LSRII Fortessa (BD Biosciences) and analysed in FlowJo (v10). The gating is shown in Supplementary Fig. 1.

### Histology

Mouse liver, kidney, lung, and colon tissue were processed, sectioned, rehydrated, and stained with Hematoxylin and eosin stain (H&E). For H&E staining, rehydrated samples were incubated in Harris’s hematoxylin for 5 min to stain the cell nuclei. Excess hematoxylin was removed by washing in tap water until clear. Slides were then stained with alcoholic eosin for 30 s and excess solution was removed with a 2 min wash in tap water. Samples were dehydrated with 100% ethanol before being air-dried and mounted in xylene. H&E samples were imaged using a ZWEISS microscope and reviewed by two pathologists.

### Immunohistochemistry

Tumour tissue was processed into 5 µm sections, which were subsequently deparaffined with xylene and then washed. Heat antigen retrieval was conducted with a Tris–EDTA buffer (pH 9.0, abcam) at 90 degrees for 1 h, followed by a 20-min incubation in 3% hydrogen peroxide to block endogenous peroxidase activity. The tissue slides were blocked with 5% goat serum (VECTASTAIN® Elite ABC Kit, Vector Laboratories, CA, USA) in 0.015% BSA and 0.01% Triton X-100 in PBS for 30 min. Tissue slides were incubated with 150 µl of PD-L1 primary antibody (1:200, Abcam) in 2% goat serum and 0.015% BSA in PBS overnight at 4 °C. Tissue slides were then washed three times with washing buffer (0.015% BSA and 0.01% Triton-100 in PBS) for 10 min each before incubation with 150 µl of secondary antibody (VECTASTAIN® Elite ABC Kit, rabbit, 1:200) in 2% goat serum and 0.015% BSA in PBS for 60 min at room temperature, followed by three washes with washing buffer for 10 min each. Visualization of cells bound to the primary antibody was achieved by incubation with diaminobenzidine chromogenic (DAB) substrate (DAB substrate kit, prepared 10 min prior to use in accordance with the manufacturer’s instructions). The slides were counterstained with Harris Haematoxylin before washing three times in 100% ethanol (3 min each) followed by three washes in 100% xylene (3 min each).

### Western blot

Cell lysates were prepared using radio immunoprecipitation assay buffer (RIPA,) and protein was quantified by Pierce™ BCA protein assay kit (Thermo Fisher #23,225). Equal amounts of protein were mixed with 5 × loading buffer and denatured at 100 °C for 5 min. The samples were loaded onto a 10% SDS-PAGE, electrophoresed at 85 V for 2 h in 1 × SDS running buffer, and then transferred to a polyvinylidene fluoride (PVDF) membrane at 100 V for 45 min. The membrane was blocked in 5% bovine serum albumin (BSA) and incubated with anti-PD-L1 antibody (1:1000, Abcam) overnight at 4 °C. The following day, the PVDF membrane was washed 3 times with 1 × TBST (Tris-Buffered Saline, 0.1% Tween 20), incubated with anti-mouse HRP-linked secondary antibody (1:10,000, Cell Signalling) for 1 h at room temperature, washed thrice with 1 × TBST, and then imaged in ChemiDoc™ Imaging System (Bio-Rad). The protein bands were quantified using ImageJ Version 1.54i.

### Statistical analysis

For a power (beta) of 0.8 and probability level (alpha) of 0.05, ten mice were used per treatment group for statistical relevance. Ordinary one-way and two-way ANOVA were performed using Graph Pad Prism version 9 software. Data were expressed as means ± standard deviation (SD) and were compared by mean of paired/unpaired student’s t test. Statistical significance was accepted at values of *p* < 0.05 and indicated in the figures by asterisks (*, *p* < 0.05; **, *p* < 0.01, ***, *p* < 0.001, ****, *p* < 0.0001).

## Results

### Combination of tislelizumab and ociperlimab resulted in partial responses in immunotherapy-naïve epithelioid PM patients

**Fig. 1:**
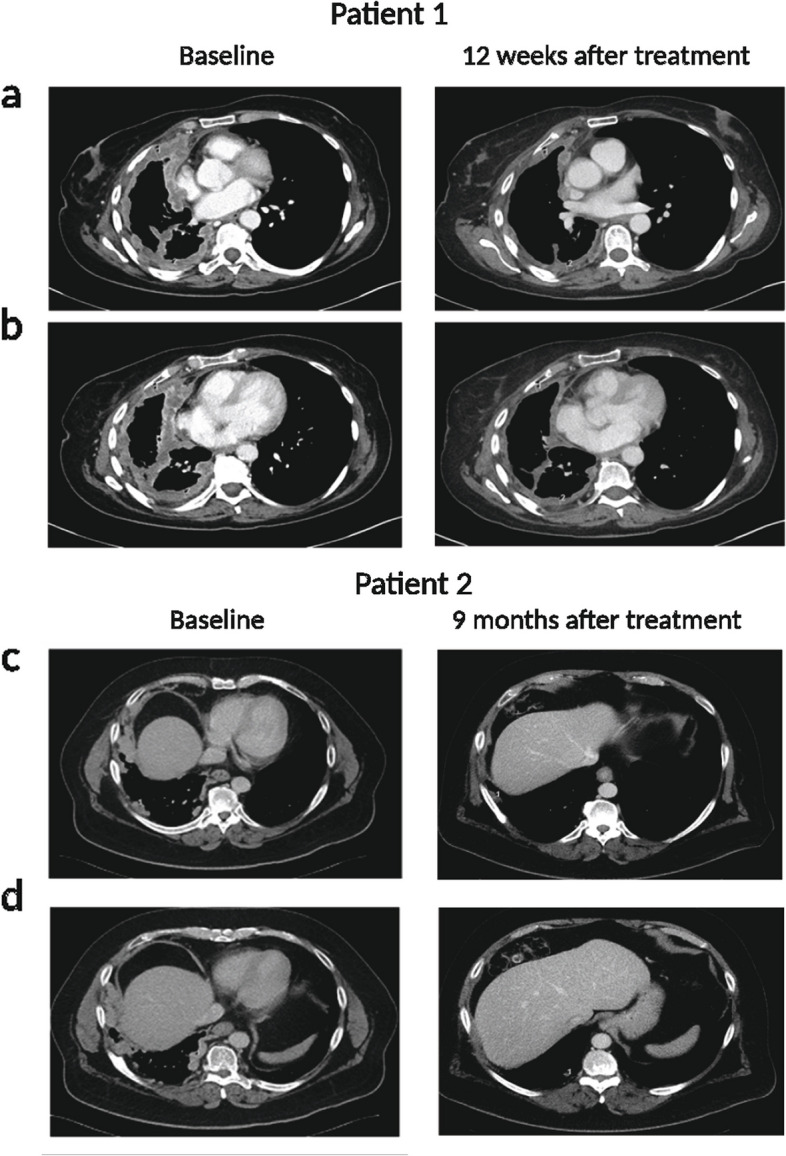
Radiological responses to tislelizumab and ociperlimab for the two PM patients. 1A and 1B represent PET-CT images of patient number one, and 1C and 1D correspond to patient number two. **A** Axial section at the level of the right superior pulmonary vein at baseline (1 = 18 mm, 2 = 10 mm) and 12 weeks after treatment (1 = 5 mm, 2 = 5 mm) (**B**) Axial section at T9 vertebra level at baseline (1 = 10 mm, 2 = 11 mm) and 12 weeks after treatment (1 = 8 mm, 2 = 8 mm). **C** Axial section at the level of right 9.^th^ rib at baseline (1 = 14 mm) and 9 months after treatment (1 = 8 mm). **D** Axial section at the level of T9 vertebra at baseline (1 = 14 mm) and 9 months after treatment (1 = 6 mm)

Two out of 32 patients enrolled in the AdvanTIG-105 study had epithelioid PM. Both patients exhibited partial responses to treatment with tislelizumab and ociperlimab. The first patient was a 58-year-old female who was diagnosed with epithelioid PM of the right lung in December 2018, characterised by loss of BAP-1 staining. There was insufficient archival tissue available for central testing of PD-L1 tumour proportional score (TPS) and TIGIT immune cell score. At the time of diagnosis, PET scan demonstrated extensive avidity throughout the right hemithorax and mediastinal lymph nodal involvement. The patient underwent 6 cycles of first-line cisplatin and pemetrexed therapy from February 2019, and stable disease was achieved in accordance with Response Evaluation Criteria in Solid Tumours (RECIST) criteria modified for mesothelioma. Treatment was withdrawn from June 2019 to August 2020 until radiological progression was observed. The patient was then recruited to the AdvanTIG-105 trial and was subjected to treatment with 200 mg of tislelizumab and 900 mg of ociperlimab every 3 weeks from October 2020. A total of 9 cycles of the treatment was administered to the patient until April 2021, with a partial response to treatment evident after 4 cycles of therapy (Fig. 1a and b). There was progressive disease in March 2021 with new metastatic deposits in the upper abdominal lymph nodes, with a progression free survival of 5.1 months. Third-line systemic therapy was given with a rechallenge of carboplatin and pemetrexed, which achieved stable disease until the patient eventually succumbed to disease progression in November 2021.

The second patient was a 65-year-old male who was diagnosed with epithelioid PM of the right lung in September 2019, with retained BAP-1 staining. Central testing of the archival sample demonstrated a PD-L1 (SP263 clone) TPS of 0% and a TIGIT (SP410 clone) immune cell score of 0%. He received first-line platinum-based chemotherapy and achieved a partial response until the occurrence of radiological progression of the right pleural-based lesion in September 2020. Joining the AdvanTIG-105 trial in October 2020, the patient underwent 15 cycles of a combination of 200 mg of tislelizumab and 1800 mg of ociperlimab every 3 weeks until October 2021, with a confirmed partial response to therapy following 6 cycles of therapy (Fig. 1c and d). Withdrawing from the trial in December 2021, he continued to have a partial response during surveillance up until evidence of radiological progression in June 2022. This patient had a progression free survival of 19.4 months for the combination immunotherapy of ociperlimab and tislelizumab.

### Anti-PD-1 and TIGIT antibodies treatment elicits a strong-tumour response in mouse epithelioid PM model with highest response and survival rate

**Fig. 2:**
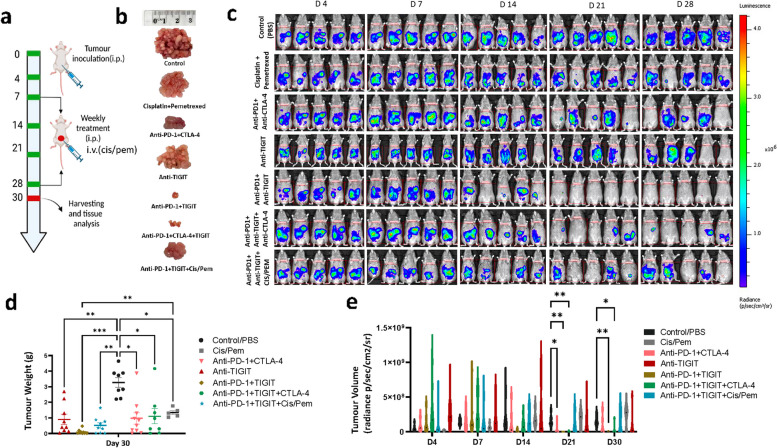
Anti-tumour effect of TIGIT immune checkpoint inhibitor in an epithelioid PM mouse model. **a** Schematic illustration of the anti-TIGIT combination treatment design to inhibit tumour growth in an epithelioid PM mouse model​. **b** Representative photographs showing the size and morphology of tumour nodules harvested at the end of the experiment (Day 30). **c** Representative in vivo bioluminescence images showing the size and distribution of tumour growth following, i.p., injection of AC29 mesothelioma cells in the mice after different treatments. **d** Average weights of tumours and **e** Tumor volume, measured by the IVIS imaging system after different treatments, is presented using violin plots to show the frequency distribution of the data. A total of ten mice were utilized per treatment group and experiments were repeated once. Error bars reflect the SD of each treatment group; statistical significances were calculated via paired t-test and two-way ANOVA, *P < 0.05, **P < 0.01

Given the responses we observed in epithelioid PM patients enrolled in the AdvanTIG-105 trial, we hypothesised that the combination of anti-TIGIT and anti-PD-1 antibodies is a more effective treatment than current standard-of-care treatment involving chemotherapy or combination immunotherapy with anti-CTLA4 and anti-PD-1 antibodies. As such, we investigated the relative efficacy of these treatment approaches using an epithelioid PM mouse model. To assess the synergistic anti-tumour efficiency of the novel integrated TIGIT blockade treatment, an AC29 PM mouse model was employed. Tumour-bearing mice were i.p. injected with different combinations of anti-PD-1, anti-TIGIT, and anti-CTLA-4 antibodies at a dose of 0.2 mg/mouse, respectively (Fig. 2a). Cisplatin and pemetrexed were administrated intravenously at doses of 0.32 mg/mouse and 2.56 mg/mouse, respectively, in conjunction with anti-PD-1, and/or anti-TIGIT antibodies. Tumour growth was monitored weekly using in vivo bioluminescence imaging (Fig. 2c).

Animals subjected to the novel treatments displayed a significant reduction in tumour weight compared to the controls (3.272 ± 0.312 g) at the conclusion of the experiment (Fig. 2b, d, e). Specifically, mice treated with the anti-PD-1 plus anti-TIGIT combination exhibited a significantly enhanced tumour suppression, resulting in a tumour weight of 0.101 ± 0.045 g, whereas those receiving standard-of-care with anti-PD-1 plus anti-CTLA-4 antibodies and cisplatin chemotherapy had larger tumour weights of 0.976 ± 0.368 g and 1.344 ± 0.290 g, respectively (Fig. 2b, d, e).

**Fig. 3:**
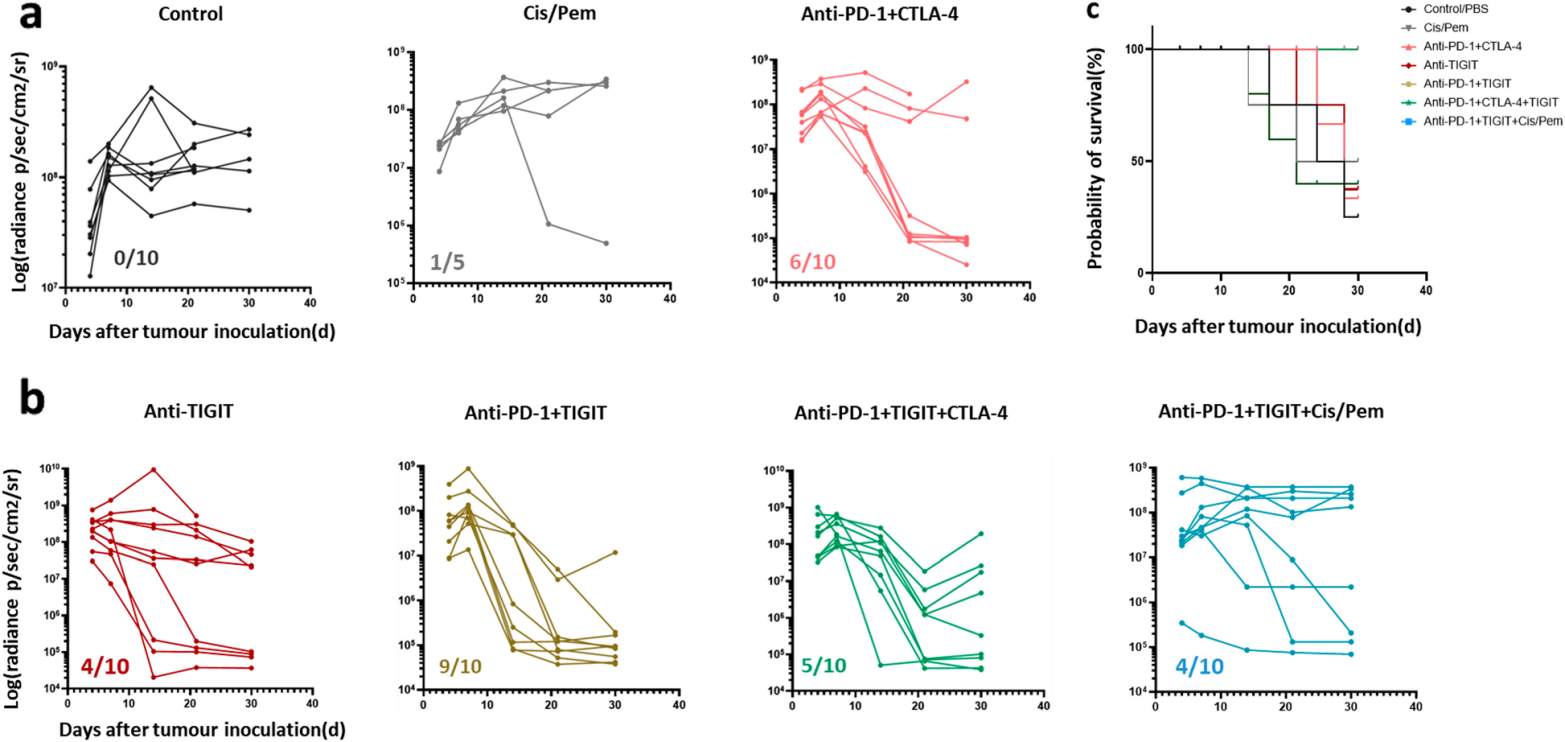
Tumour volume measured by the IVIS imaging systems after different treatments. In vivo tumour growth and immunotherapy responses of individuals after **a**) standard care and **b**) novel TIGIT treatments. Anti-PD-1 single-armed treatment was presented in supplementary Fig. 5. Numbers indicate tumour-regression animals/total animals at completion. **c** Overall survival at the end of treatments (Day 30). *N* = 5–10 mice were utilized per treatment group

Moreover, the combination treatment with anti-PD-1 plus anti-TIGIT antibodies demonstrated a robust anti-tumour response, resulting in a 90% response rate and 100% survival rate at the time of harvesting. This result was recapitulated by the anti-PD-1 plus anti-CTLA-4 regimen, with a response rate of 60% and survival rate of 90% (Fig. 3a). Conversely, treatment with anti-TIGIT alone (with a response rate of 40%, and a survival rate of 70%) or anti-TIGIT in combination with anti-PD-1 plus anti-CTLA-4 (with a response rate of 50%, and a survival rate of 50%) or anti-TIGIT in combination with cis/pem chemotherapy (with a response rate of 40%, and a survival rate of 50%) failed to elicit synergistic effects that could further enhance their anti-tumour potency (Fig. 3b). Notably, bioluminescence imaging revealed a significant reduction in tumour volume after anti-PD-1 plus anti-TIGIT antibody treatment on Day 21 and Day 30 compared to the controls treated with PBS (Fig. 2e). Strikingly, all mice responded to treatment with PD-1 and TIGIT co-blockade, with 9 out of 10 mice remaining tumour-free for over 300 days (Fig. 3c).

### *Anti-PD-1 and TIGIT antibodies treatment activates tumour infiltrated T reg, CD107a* + *T cells and NK cells*

**Fig. 4:**
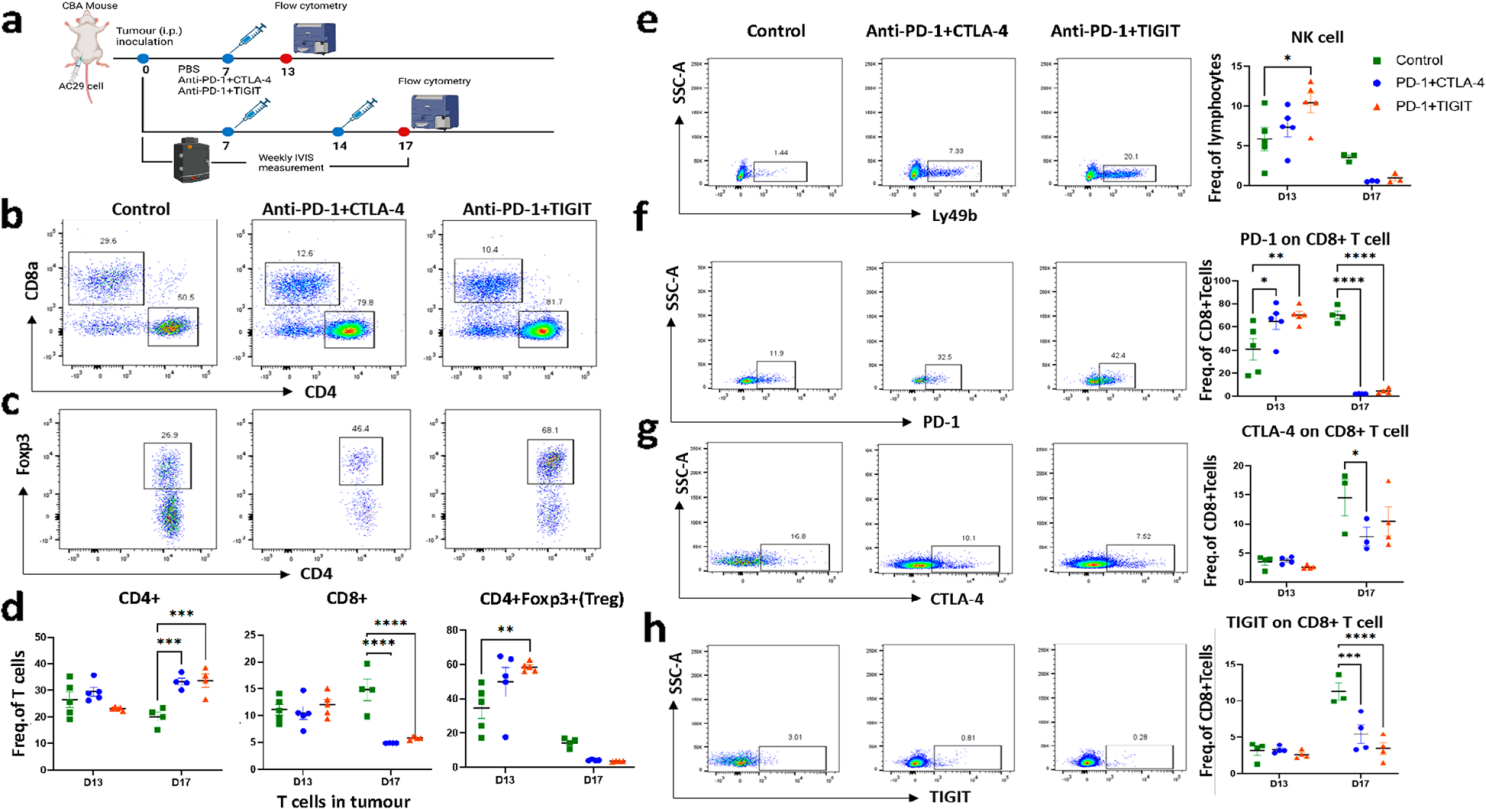
Anti-PD-1 plus anti-TIGIT combination therapy activating systematic anti-tumour immunity. **a**, **b**, **c** Representative flow-cytometry plots (Day 13) showing the tumour-infiltrating CD4 + , CD8 + , CD4 + FoxP3 + (Treg) cells. **d** Enumeration of CD4 + , CD8 + , T reg cells in tumour. Representative flow-cytometry plots and relative percentage of **e**) NK cells and **f**) PD-1, **g** CTLA-4, **h** TIGIT expression on CD8 + T cells in tumour. *N* = 4–5 mice were used per group and per timepoints. Error bars reflect the SD determined from each treatment group. Statistical significances were calculated by two-way ANOVA. * *P* < 0.05, **P < 0.01, ***P < 0.01, ****P < 0.0001

To understand the underlying mechanisms driving the enhanced anti-tumour response induced by treatment with the anti-PD-1 and anti-TIGIT antibody combination, an additional mouse experiment was conducted to monitor infiltrations of T and NK immune cells. An analysis of immune cells in the spleen and tumour were carried out using flow cytometry at two time points; one following the first treatment (Day 13) and the other following the second treatment (Day 17) (Fig. 4a). No substantial alterations were observed in the sphenic immune cell population (Supplementary Fig. 2), however the results revealed a notable elevation in the proportion of CD4 + T cells within the tumour following the second treatment; both with anti-PD-1 plus CTLA-4 (33.25 ± 2.79%) and anti-PD-1 plus TIGIT (33.60 ± 5.02%), when compared with the PBS-treated controls (20.03 ± 3.55%). Furthermore, the proportion of CD4 + /Foxp3 + regulatory T cells and NK cells in animals treated with anti-PD-1 plus TIGIT antibodies were 58.50 ± 2.75% and 10.40 ± 2.71% respectively; exhibiting a significant 50% increment compared with the PBS control (34.46 ± 13.35%; 5.85 ± 3.21%), or a 30% surge relative to anti-PD-1 plus CTLA-4 antibodies treatment (49.70 ± 19.05; 7.31 ± 2.60%) at Day 13 (Fig. 4 d,e). More importantly, animals treated with anti-PD-1 and TIGIT antibodies showed an increase in immune-infiltrated lymphocytes per tumour weight as well as the number of CD107a + CD8 + T cells and their ratio to CD4 + /Foxp3 + regulatory T cells within the tumour (Supplementary Fig. 3, 4). This suggests an enhanced immune stimulation response from activated cytotoxic T cells. The expression of immune-checkpoint inhibitors PD-1, CTLA-4 and TIGIT were all significantly downregulated on CD8 + T cells following the second treatment at Day 17 (Fig. 4 f, g, h). Interestingly, animals subjected to anti-PD-1 plus TIGIT treatment displayed the highest PD-1 expression on CD8 + T cells when compared to those treated with the anti-PD-1 plus CTLA-4 combination or untreated control animals (Fig. 4f).

### *Anti-PD-1 and TIGIT antibodies treatment increased CD8* + *effector cells and enhanced long term immune memory*

**Fig. 5:**
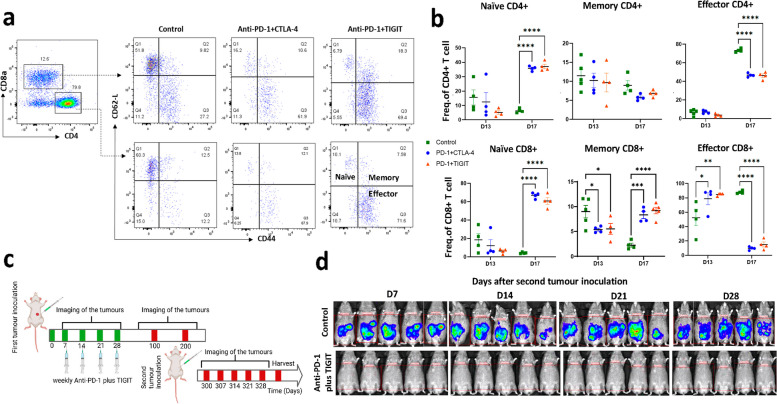
Effects of Anti-PD-1 plus TIGIT combination therapy further enhanced tumour CD8 + effector and memory T cell activation. **a** Representative flow-cytometry plots of tumour naïve (Q1), memory(Q2) and effector(Q3) CD4 + (bottom row) and CD8 + (top row) T cells in tumour **b**) Relative percentages of naïve CD4 + /CD8 + , effector CD4 + /CD8 + and memory CD4 + /CD8 + cells in tumour. ​**c** Schematic illustration of the experiment design to assess the immunological memory response triggered by Anti-PD-1 plus TIGIT combination therapy. **d** Representative in vivo bioluminescence images showing the size and distribution of tumour growth following the second inoculation of AC29 tumour cells at Day 300 without any treatment. Tumour naïve mice were used as control (*N* = 5). Statistical significances were calculated by two-way ANOVA. *N* = 4–5 for flow cytometry, *N* = 5–9 for tumour rechallenge. * *P* < 0.05, **P < 0.01, ****P < 0.0001

Subsequently, we delineated distinct T cell subtypes in the tumour by flow cytometry, including naïve, effector and memory CD4 + /CD8 + T cells following the initial and second anti-PD-1 combination therapies (Fig. 5a). Animals treated with second dose of either anti-PD-1 plus CTLA-4 or anti-PD-1 plus TIGIT antibodies demonstrated a significant increase in the naïve CD4 + /CD8 + population on Day 17 compared to the PBS-treated controls (Fig. 5b). The proportion of effector CD8 + T cells reached its zenith in animals treated with anti-PD-1 plus TIGIT antibodies, measuring 84.83 ± 1.87% on Day 13, in contrast to 78.85 ± 16.53% for anti-PD-1 plus CTLA-4 and 52.48 ± 22.66% for the PBS controls (Fig. 5b). By Day 17, the proportion of CD4 + /CD8 + effector T cells significantly decreased in tumours treated with antibody (Fig. 5b).

Immunological memory response, a recognised hallmark of the adaptive immunity, plays a pivotal role in safeguarding organisms against subsequent pathogen incursions. In this context, we observed an amplified CD8 + memory T cell response after the second dose of anti-PD-1 plus TIGIT antibodies treatment, measuring 10.05 ± 0.91%. This contrasted with 8.31 ± 1.40% for animals treated with anti-PD-1 plus CTLA-4 and 2.25 ± 0.83% for the PBS-treated controls (Fig. 4b). This led us to further investigate the immune memory effects of our combined anti-PD-1 plus anti-TIGIT antibody therapy. This was pursued by rechallenging mice on Day 300, which was 270 days post initial anti-PD-1 plus anti-TIGIT antibody treatment (Fig. 5c). Simultaneously, five control mice were inoculated with the same number of AC29 cells for comparative assessment. The results indicated that tumour growth upon rechallenge was substantially suppressed in the combined treatment group compared to the control group (Fig. 5d). Impressively, all mice (9/9) subjected to the combined treatment demonstrated resistance to rechallenge, while control mice exhibited tumour development and succumbed within 30 days post-inoculation. These compelling findings underscore the generation of enduring immune-memory effects induced by anti-PD-1 plus anti-TIGIT blockade treatment.

### Anti-PD-1 and TIGIT antibodies treatment reduces PD-L1 expression

**Fig. 6:**
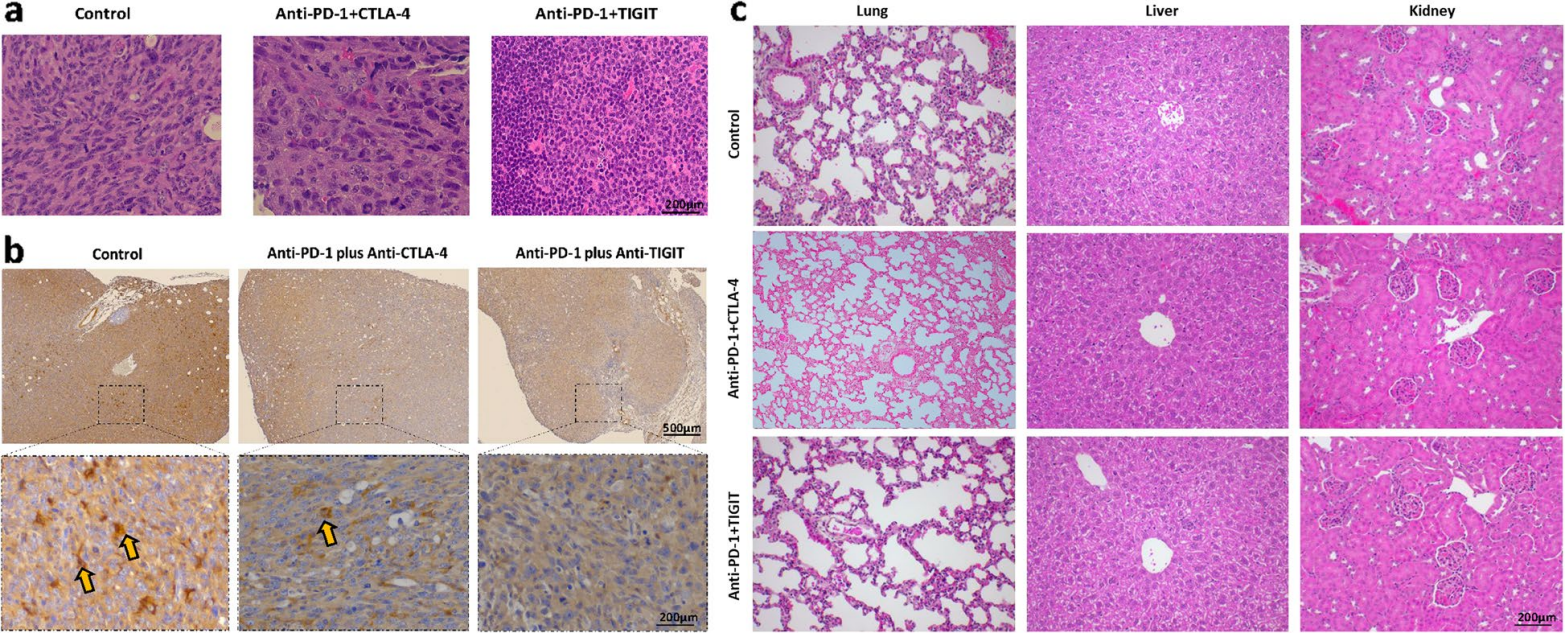
Anti-PD-1 plus TIGIT combination therapy reduced the tumoral PD-L1 expression without any observed histological adverse effects. **a** Representative H&E staining of tumour at day 30 post tumour inoculation. **b** representative IHC staining of PD-L1 expression in tumour under different treatments. Yellow arrows indicate PD-L1 positive tumour cells. **c** Representative H&E staining of lung, liver and kidney harvested at the end of combination checkpoint blocked immunotherapy

A western blot of mouse epithelioid mesothelioma AC29 cells was conducted to confirm PD-L1 expression before tumour inoculation (Supplementary Fig. 4). Post immunotherapy, we continue to observe high level of PD-L1 expression in tumours. Additionally, there is a high level of immune cell infiltration in the anti-PD-1 + TIGIT-treated tumour tissue sections with a decreased level of PD-L1 expression when compared to anti-PD-1 + CTLA-4-treated or PBS control-treated tumour tissue sections (Fig. 6 a,b).

Microscopic examination and scoring of 15 lung, liver and kidney tissue sections, obtained post immunotherapy, did not show evidence of immune-related adverse effects. All examined organs were found to exhibit normal histological features without inflammation, immune cell infiltration, or other pathological changes associated with immunotherapy-related reactions (Fig. 6c).

## Discussion

The current FDA-approved combination treatment of PD-1 and CTLA-4 has been proven to extend mesothelioma patient survival beyond those treated with chemotherapy. However, the substantial side effects of this combination, coupled with a response rate of only about 40%, have prompted us to explore better treatment options. TIGIT, a co-inhibitory receptor regulating T cell function, negatively modulates T cell responses when interacting with its ligands (CD155/CD122), making it an attractive target for immunotherapy.

To the best of our knowledge, the outcomes of our mouse study, illustrating the remarkable 90% response rate and the lack of immune-mediated adverse events for the combination of anti-PD-1 and anti-TIGIT antibodies, coupled with the sustained tumour immunity even after 300 days of tumour rechallenge, constitute a promising advancement in the realm of immune checkpoint combinations. This preclinical research represents the inaugural demonstration of such promising synergistic effects. This is further corroborated by our clinical observation in two epithelioid PM patients achieving partial responses following treatment with tistlelizumab and ociperlimab, a combinational anti-PD-1 and anti-TIGIT antibody therapy administered as part of a phase 1 trial protocol. The partial responses were durable, and the response was sustained for eight months after cessation of tislelizumab and ociperlimab in one patient. We believe that this is the first clinical report of epithelioid PM patients treated with this combination of therapy in the literature [14].

Given the remarkable responses observed in the animal model treated with anti-PD-1 and anti-TIGIT antibodies, we were interested in determining the mechanism of the increased tumour response associated with this combination treatment. Our results indicated a significant increase in CD4 + and CD8 + T cells in the tumour micro-environment, rather than peripherally following treatment with anti-PD-1 and anti-TIGIT. This enhancement of tumour infiltrated lymphocytes (TILs) reflects the immune system’s attempt to eliminate tumour cells. A higher frequency of infiltrated activated CD8 + effector T cells and NK cells were associated with better tumour suppression compared to the control or other treatment groups. This suggests that a CD8 + T cell-mediated anti-tumour response is potentiated by the combination of anti-PD-1 and anti-TIGIT treatment.

At Day 13, a week after the first dose, this data revealed that there was not yet an increase in T cells and NK cells in treated tumours compared to untreated tissue, although there was already an increase in T reg and NK cells in treated animals. By Day 17, a few days after the second treatment, flow cytometry detected a dramatic increase in the proportion of CD4 + T cells in the tissues of mice treated with anti-PD-1/TIGIT and anti-PD-1/CTLA4. Collectively, these data suggest that the animals responded well to the combination treatment. There was an increase in CD8 + T cells and NK cells, indicating potential anti-tumour activity.

Our study demonstrates a significant increase in CD8 + memory T cell tumour infiltration in response to the combination of anti-PD-1 and anti-TIGIT, especially after the second dose, where the memory T cell population reaches its highest level. This increased level of memory CD8 + T cells likely lead to total tumour rejection in animals and resistance to tumour growth after rechallenging on Day 300. Our results suggest that memory CD8 + T cells “remember” tumour antigens, leading to potent immune reactions that suppress tumour regrowth in all challenged animals previously treated with anti-PD-1 and anti-TIGIT antibodies.

Tumour-infiltrating lymphocytes, including CD8 + T cells, CD4 + helper T cells, and NK cells, form a critical part of the tumour microenvironment [16, 17]. High densities of activated TILs within tumours are often correlated with improved prognosis, reflecting the immune system’s efforts to counteract tumour growth. Effective therapies aim to enhance the recruitment and activation of TILs, creating a favourable immune milieu for sustained anti-tumour responses. Further enrichment of TILs in the tumour microenvironment was observed in animals treated with anti-PD-1 and anti-TIGIT contributing to the robust response rate and long-term overall survival.

The availability of biomarkers able to predict favourable responses is critical to progress this work to a Randomised Clinical Trial (RCT). This could potentially allow rapid translational research using blood samples from prospective patients treated with the anti-PD-1 and anti-TIGIT antibodies. We therefore assessed the potential of splenic immune cell populations as biomarkers in this model. Unfortunately, there were no significant differences in immune cells (CD4 + , CD8 + , Treg, NK) and checkpoint inhibitors (PD-1, CTLA-4, TIGIT) found between anti-PD-1/TIGIT and anti-PD-1/CTLA-4 combination immunotherapy (Supplementary Fig. 2). BAP1 staining is a routine procedure for patients with mesothelioma and has been extensively studied as a tumour suppressor in this context. However, we did not observe any correlation between BAP1 expression and clinical response, as we only had data from two patients. In the case of renal cell carcinoma, there is limited evidence suggesting that BAP1 mutations may be associated with improved progression-free survival when patients are treated with immunotherapy, as opposed to targeted therapies [18]. Conversely, a single-institution study on mesothelioma indicated that BAP1 does not have an impact on the response to immune checkpoint inhibitors or on disease progression [19]. Therefore, it is essential to further investigate BAP1 expression levels in tumor samples from mesothelioma patients.

It should be acknowledged that there have been several negative trials involving the use of anti-TIGIT antibodies. SKYSCAPER-01 – a phase III trial of tiragolumab (anti-TIGIT antibody) and atezolizumab (anti-PD-L1 antibody) in unresectable stage 4 NSCLC had a negative co-primary endpoint of PFS, while the phase III trial SKYSCRAPER-02 also failed to find PFS or OS benefits from Tiragolumab in patients with extensive-stage small cell lung cancer (SCLC) [20, 21]. Notably, these trials were conducted without any prior preclinical studies. The absence of preclinical data makes it difficult to evaluate why the particular antibodies utilised in these trials failed in the first place. Hence, this highlights the importance of comprehensive testing of novel immune checkpoint inhibitors in preclinical in vivo models, as exemplified by our study.

Despite the negative studies, there have been a cascade of positive anti-TIGIT early phase clinical trials across multiple tumour types, indicating the ongoing potential of targeting this immune checkpoint. The recent phase II CITYSCAPE-02 trial showed that the combination of Tiragolumab (anti-TIGIT antibody) and atezolizumab (anti-PD-L1 antibody) significantly improved the objective response rate (ORR) and progression-free survival (PFS) in non-small cell lung cancer (NSCLC) patients compared to a placebo [21, 22]. The Phase 1a/1b trial across tumour types showed preliminary anti-tumour activity in NSCLC and oesophageal cancer [23]. The randomised Phase Ib/I MORPHEUS-Liver trial in 58 patients with unresectable hepatocellular carcinoma found that tiragolumab added to atezolizumab and bevacizumab had an ORR of 42.% compared to 11.1% in the control arm and a PFS hazard ratio of 0.42 (95%CI 0.22–0.82) [24]. Meanwhile, the recently reported Phase 2 EDGE-Gastric assessed domvanalimab (anti-TIGIT antibody) and zimberelimab (anti-PD-1 antibody) with and without chemotherapy in 40 patients with first-line unresectable gastric, gastroesophageal junction or oesophageal adenocarcinoma. An ORR of 80% was found in PD-L1 high tumours and 59% overall, while PFS data remains immature [25]. The ARC-7 Phase-II trial used the same agent in untreated, unresectable PD-L1-high NSCLC with improvements in median PFS and ORR observed [26]. Phase II-III trials are ongoing in multiple solid tumour types, with agents of interest including domvanalimab (ARC-10 [27]), ociperlimab (AdvanTIG-205 [28]), vibostolimab (MK-7684 [29]) and tiragolimab (IMbrave152 [30]). The ongoing pan-tumour investigation into anti-TIGIT efficacy and the promising translational research reported here in mesothelioma, suggests that TIGIT remains a potentially highly promising target. Furthermore, TILS isolated from patients with PM express significantly higher levels of TIGIT compared to tumour-free lung tissue [31]. This lends further support of conducting a trial in mesothelioma using the TIGIT immunotherapeutic approach.

The results of this study have significant implications for advancing therapeutic strategies in the field, particularly given the superiority of the combined anti-PD-1 and anti-TIGIT blockade in the epithelioid mesothelioma animal model, compared to the clinical standard-of-care treatments such as cisplatin/pemetrexed chemotherapy [32] and anti-PD-1 and anti-CTLA-4 inhibition [6]. The outcomes provide the robust foundation for the development of a RCT aimed at evaluating the comparative efficacy and toxicity of the combined anti-PD-1 and anti-TIGIT treatment to nivolumab and ipilimumab in patients with unresectable epithelioid mesothelioma. This breakthrough could potentially revolutionize treatment paradigms for this challenging patient population.

## Conclusion

Our study presents evidence of the potent synergy achieved through the combination of anti-PD-1 and anti-TIGIT antibodies, leading to a remarkable 90% response rate, complete tumour rejection, and the establishment of enduring tumour immunity, in this mouse model of epithelioid mesothelioma. Importantly, the efficacy is superior compared to the clinical standard-of-care treatments such as chemotherapy and combination immunotherapy of anti-PD-1 and anti-CTLA-4 antibodies. This is supported by the clinical observation of two epithelioid mesothelioma patients who had durable partial responses treated with tislelizumab and ociperlimab. These findings present a robust impetus for further research, prompting us to advocate for the initiation of a randomised clinical trial to translate these promising results into tangible clinical benefits for patients with unresectable mesothelioma.

## Supplementary Information

Supplementary Material 1.

## Data Availability

All data generated that are relevant to the results presented in this article are included in this article. Other data not relevant to the results presented here are available from the first author, Dr. Shi, upon reasonable request.

## Abbreviations

PM: Pleural mesothelioma
TIGIT: T cell immunoreceptor with Ig and ITIM domains
NK: Natural killer cells
Treg: Regulatory T cells
ICIs: Immune checkpoint inhibitors
H&E: Hematoxylin and eosin stain
TPS: Tumour proportional score
RECIST: Response Evaluation Criteria in Solid Tumours
TILS: Tumour infiltrated lymphocytes
RCT: Randomised Clinical Trial
SCLC: Small cell lung cancer
ORR: Objective response rate
PFS: Progression-free survival
NSCLC: Non-small cell lung cancer
