# Supplementary material for: A combination of PD-1 and TIGIT immune checkpoint inhibitors elicits a strong anti-tumour response in mesothelioma
Source: J Exp Clin Cancer Res. 2025 Feb 12;44:51. doi: 10.1186/s13046-025-03314-w (PMC11816573; doi:10.1186/s13046-025-03314-w)
Supplement: Supplementary file 1 — Supplementary Material 1. [file 13046_2025_3314_MOESM1_ESM.docx]

**Supplementary Data**


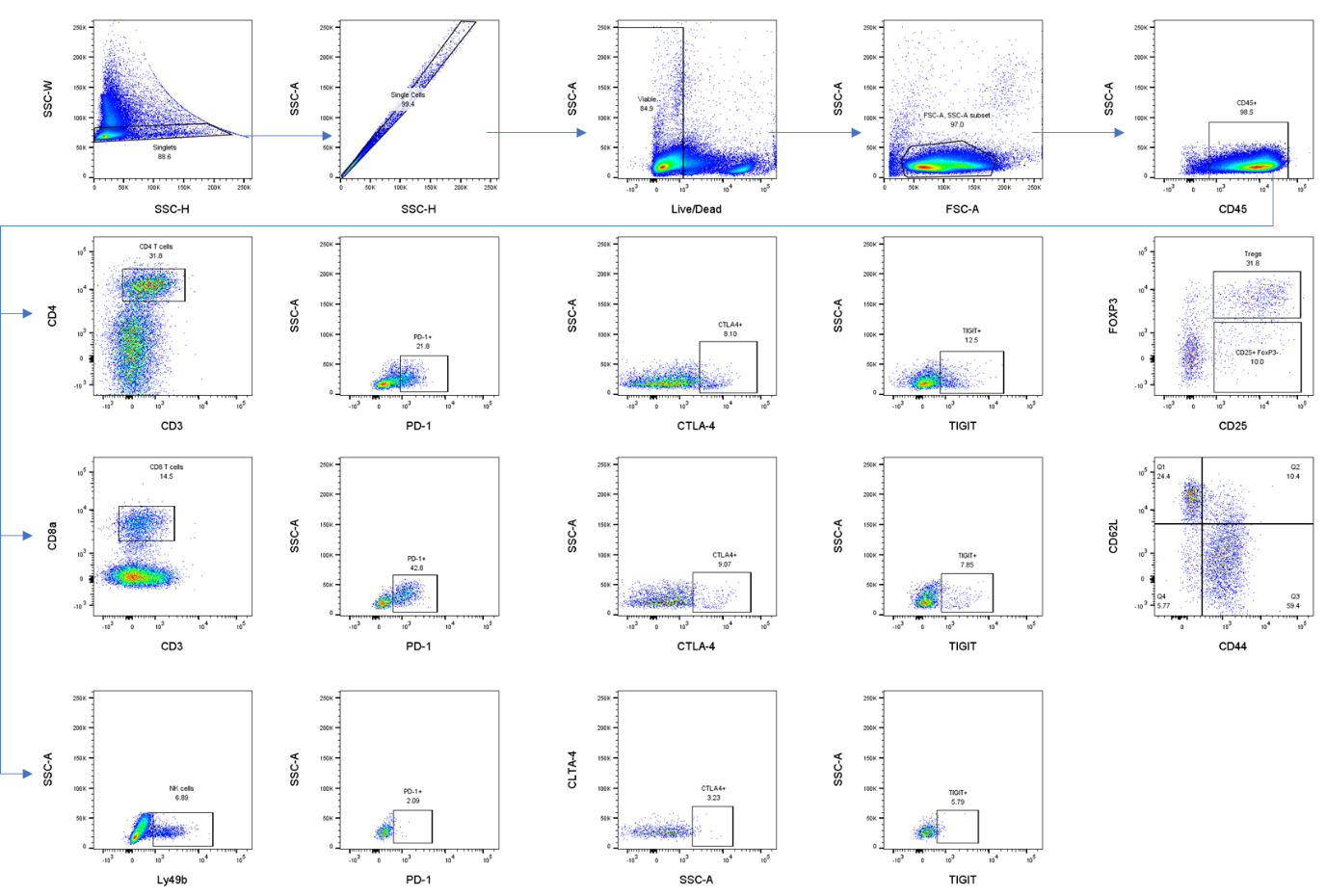


**Supplementary Figure 1.** Flow cytometry Gating Strategy. This strategy was applied in main figures 3, 4 and supplementary figure 2. Cells were first gated on SSC-A and SSC-H to define single cells. Then, gate live cells, CD45+ cells, CD4+ cells, CD8+ cells and Ly49b+ cells. PD-1+, CTLA-4+, TIGIT+, and Foxp3+ cells were also gated according.

**
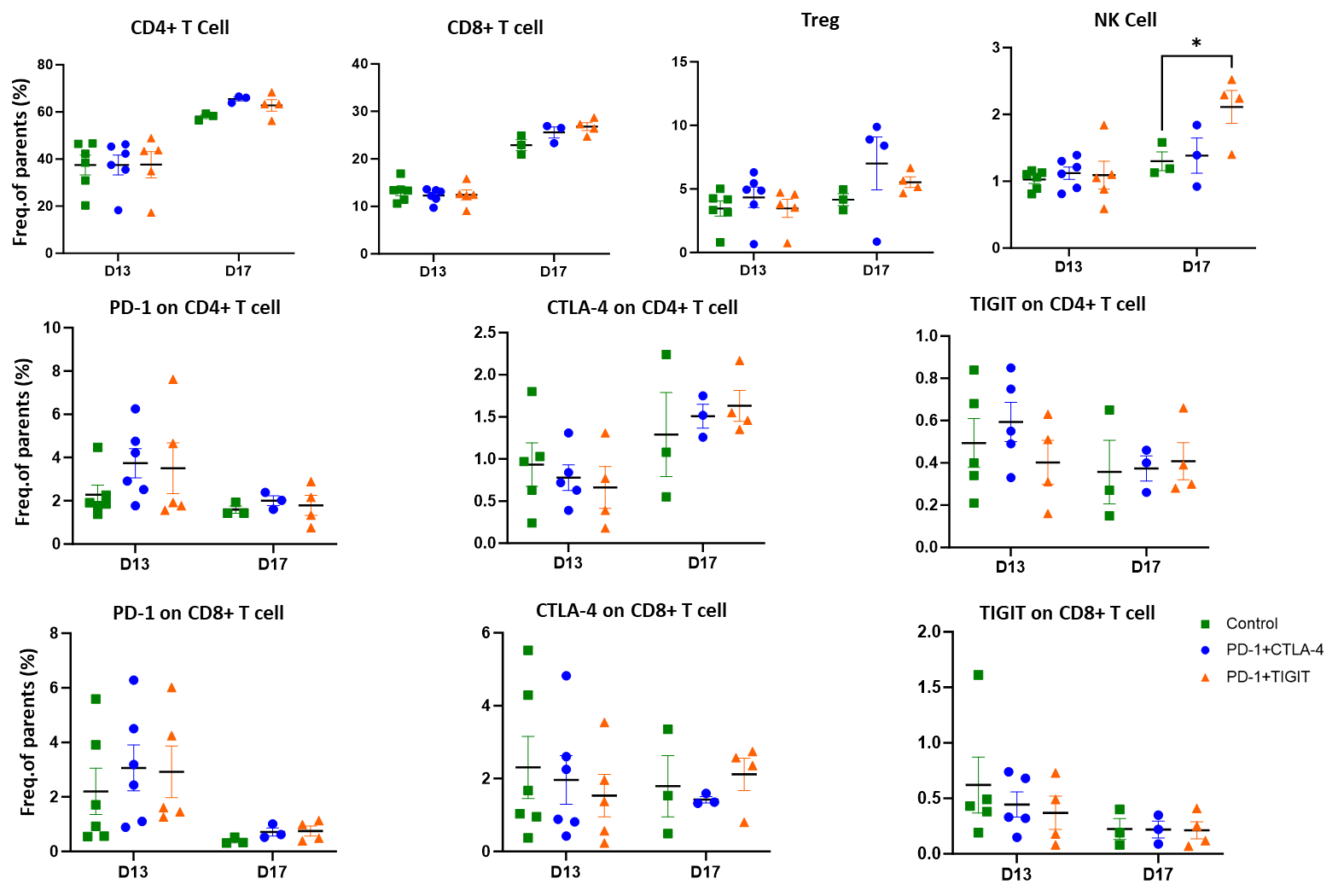
**


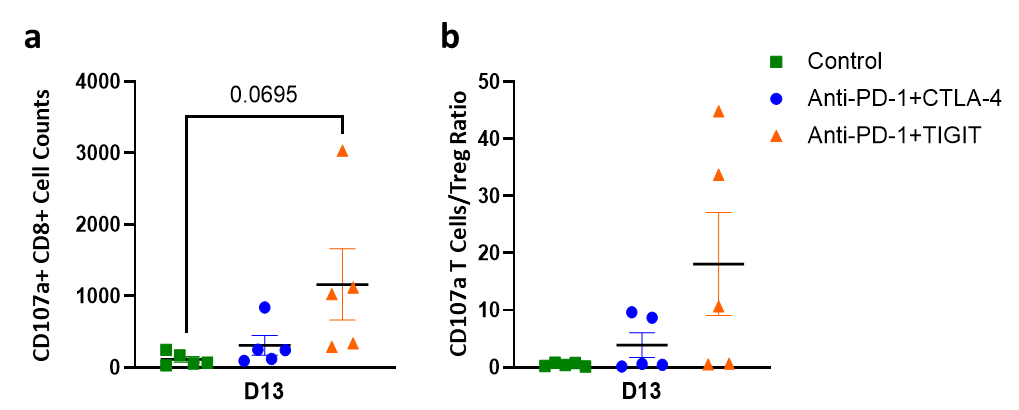
**Supplementary Figure 2.** Quantifying CD4+, CD8+, T reg, NK cells and PD-1, CTLA-4 in proportion to parent cell CD45+ (%). TIGIT expression on T cells in the spleen. Anti-PD-1+anti-TIGIT treatment demonstrated an elevation in NK cells; otherwise, no significant differences were observed. N=4-5 per group per time point. Statistical significances were calculated by two-way ANOVA. * P<0.05.

Supplementary Figure 3. Anti-TIGIT-treated tumours demonstrated an increase in CD107a+ CD8+ T cells (a) and the ratio between CD107a T cells and Tregs (b), N=4-5; error bars reflect the SEM of each treatment group.


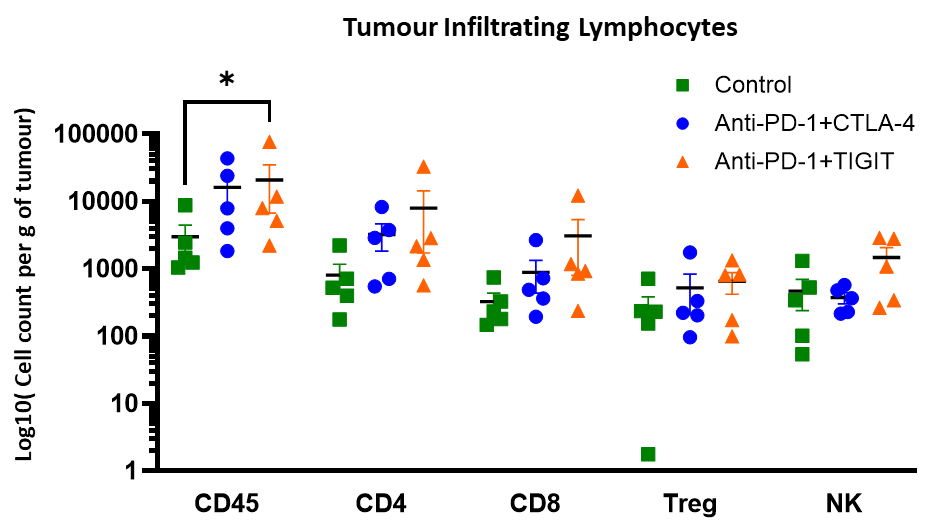


Supplementary Figure 4. Anti-PD-1+TIGIT treated tumours showed increased infiltrating lymphocytes per tumour weight. N=5, error bars reflect the SEM of each treatment group.


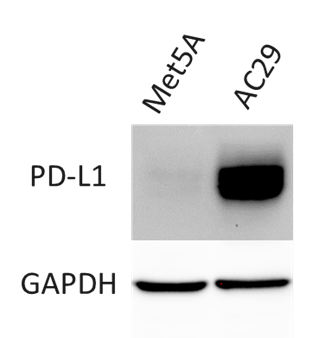

**Supplementary Figure 5**. PD-L1 expression on mouse epithelioid mesothelioma cell line AC29 was confirmed by western blot. Non-malignant mesothelium cell Met5A was used as a control.

**Anti-PD-1 (200 μg/mouse, RMP1-14)**

**D28**

**D21**

**D14**

**D7**


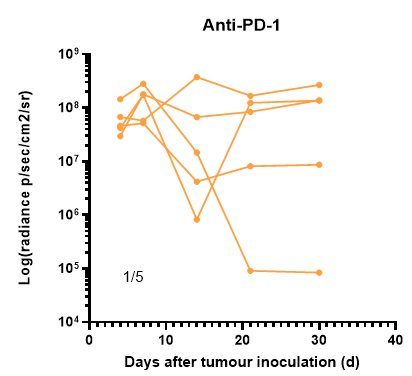

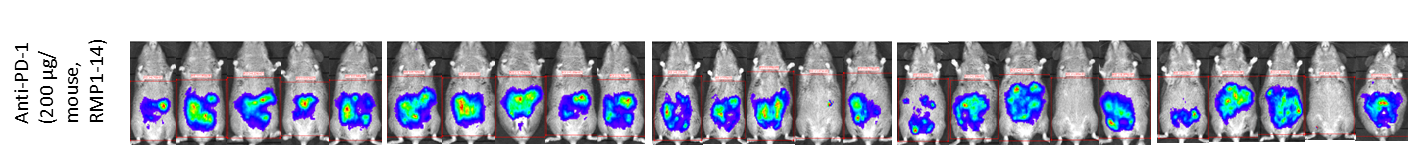


**Supplementary Figure 6.** Tumour volume was measured by the IVIS imaging systems after an anti-PD-1 single-armed treatment.

**Supplementary Table 1. Antibodies used for immunotherapy treatments**

| **Antibody** | **Name** | **Source** | **Reacts with** | **Reported Applications** |
| --- | --- | --- | --- | --- |
| Anti-PD-1 | *InVivoPlus anti-mouse PD-1 (CD279)*  ***(#BP0146)*** | BioXcell | mouse PD-1 (programmed death-1) | *in vivo* blocking of PD-1/PD-L signalling |
| Anti-CTLA-4 | *InVivoPlus anti-mouse CTLA-4 (CD152)*  ***(#BP0131)*** | BioXcell | Mouse CTLA-4-human IgG1 fusion protein | *in vivo* CTLA-4 neutralization |
| Anti-TIGIT | *InVivoPlus anti-mouse TIGIT (****#BP0274)*** | BioXcell | Mouse TIGIT  (T cell immunoreceptor with Ig and ITIM domains) | *in vivo* TIGIT stimulation |
